# Unmasking Pulmonary Nocardiosis in an Asthmatic Host Presenting With Chronic Cough, Pulmonary Nodularity, and Ground-Glass Opacities

**DOI:** 10.7759/cureus.84739

**Published:** 2025-05-24

**Authors:** Marie Yung-Chen Wu, Thomas C Tsai, Chih-Heng Hsieh, Felipe Barbosa, Aba Somers

**Affiliations:** 1 Internal Medicine, MetroWest Medical Center, Tufts University School of Medicine, Framingham, USA; 2 Infectious Diseases, MetroWest Medical Center, Tufts University School of Medicine, Framingham, USA; 3 Pulmonary and Critical Care Medicine, MetroWest Medical Center, Tufts University School of Medicine, Framingham, USA

**Keywords:** asthma, bronchoalveolar lavage, bronchoscopy, lung nocardiosis, nocardia nova

## Abstract

A 53-year-old man was evaluated in the pulmonary clinic for ongoing asthma symptoms. Despite optimized treatment, he continued to experience a chronic productive cough. He was otherwise physically active, a lifelong non-smoker with no known occupational or environmental exposures. Pulmonary function tests demonstrated normal diffusing capacity and mild airflow obstruction. A chest computed tomography (CT) scan revealed basilar bronchiectasis, mediastinal lymphadenopathy, scattered nodules, and progressive ground-glass opacities (GGOs) in the bilateral lower lobes. Immunoglobulin (Ig) testing showed an isolated IgG3 deficiency, while other Ig subclasses were within normal limits. Additional laboratory workup, including alpha-1 antitrypsin levels, antinuclear antibody, and HIV serologies, was unremarkable. Bronchoscopy was performed, and bronchoalveolar lavage (BAL) cultures grew pan-sensitive *Pseudomonas aeruginosa*, prompting a course of levofloxacin. Despite an initial negative acid-fast bacilli (AFB) stain, cultures later identified *Nocardia nova*. Given this diagnosis, a head CT was obtained to assess for central nervous system involvement, which was negative. The patient was initially started on trimethoprim-sulfamethoxazole but developed an adverse reaction, necessitating a switch to an extended course of intravenous ceftriaxone, which was later changed to clarithromycin based on susceptibility testing. A repeat chest CT demonstrated resolution of nodularity, GGOs, and lymphadenopathy. His symptoms fully resolved, with no recurrence of cough or asthma-like exacerbations. Although *Nocardia* infections are commonly associated with immunosuppression, typically occurring in patients with solid organ or hematopoietic cell transplants, chronic glucocorticoid use, malignancy, or HIV with low CD4 counts, approximately one-third of cases are reported in immunocompetent individuals. While cellular immunocompromise is a well-established risk factor, isolated IgG3 deficiency is not. Asthma, particularly when treated with corticosteroids or accompanied by structural lung disease, is also recognized as a key predisposing respiratory condition. Pulmonary involvement is most frequent, though dissemination to the central nervous system and skin can also occur. This case underscores the need to consider pulmonary nocardiosis even in patients without an overtly immunocompromised state who present with unexplained respiratory symptoms. Diagnosis requires a high index of clinical suspicion and may necessitate bronchoscopy or other invasive procedures when initial treatments fail. Prompt identification and targeted antimicrobial therapy are essential for a favorable outcome.

## Introduction

Nocardiosis is an infection caused by *Nocardia* species, which are aerobic, gram-positive, weakly acid-fast, branching filamentous bacteria commonly found in soil and decaying organic matter. The infection can manifest as cutaneous, pulmonary, or central nervous system (CNS) disease [1]. Pulmonary nocardiosis typically manifests as a subacute or chronic disease with symptoms such as cough, dyspnea, hemoptysis, fever, and systemic signs of illness [2]. However, its nonspecific presentation frequently mimics other respiratory diseases, leading to delays in diagnosis and treatment. Nocardiosis is most commonly seen in immunocompromised patients, including those undergoing solid organ or hematopoietic cell transplantation, individuals receiving chronic glucocorticoid therapy, and patients with HIV/AIDS, malignancy, alcoholism, or diabetes mellitus [3-5]. Epidemiologic studies suggest that up to one-third of cases occur in immunocompetent individuals [1].

While cellular immunodeficiency is a well-established risk factor, no selective immunoglobulin or IgG subclass deficiency has been definitively linked to an increased risk of nocardiosis [6,7]. Diagnostic challenges arise from nocardiosis's nonspecific clinical features, slow-growing cultures, and frequent misidentification. In many cases, invasive procedures such as bronchoscopy or lung biopsy are required to establish a definitive diagnosis, particularly when noninvasive methods are inconclusive [3].

This article was previously presented as a meeting abstract at the 2023 CHEST Annual Meeting on October 10, 2023 [8].

## Case presentation

A 53-year-old male patient with a history of mild persistent asthma, atopic rhinitis, nasal polyps, and well-controlled type 1 diabetes mellitus (T1DM) presented to the pulmonary clinic with a chronic cough.

The patient, a lifelong non-smoker with no pets and no known occupational or environmental exposures, was referred to the pulmonary clinic for better asthma control. He typically experienced acute asthma exacerbations once or twice a year, triggered by recurrent sinus infections and pneumonias, which were managed with antibiotics and short courses of glucocorticoids. However, over the past seven months, despite appropriate maintenance therapy, including a fluticasone-vilanterol inhaler, montelukast, and albuterol used only prior to exercise, he developed a persistent, non-bloody, productive cough. The sputum volume gradually increased and was most prominent in the mornings. He denied dyspnea at rest or with exertion, as well as fever, chills, weight changes, night sweats, or skin changes.

He was afebrile, normotensive, and exhibited a normal heart rate; additionally, his oxygen saturation was 94% on room air. Pulmonary examination revealed no crackles or wheezing. Skin and neurological examinations were unremarkable. Laboratory testing, including a complete blood count and basic metabolic panel, was largely unremarkable, except for a mildly elevated random glucose level of 154 mg/dL. Alpha-1 antitrypsin, antinuclear antibody, and rheumatoid factor were within normal limits. Given the prolonged cough, the infectious workup was expanded to assess for underlying immunodeficiency. An immunoglobulin panel revealed a decreased IgG3 level of 8 mg/dL (reference range: 15-102 mg/dL), while other IgG subclasses and total immunoglobulins were within normal limits. HIV-1/2 antibodies were negative. Sputum cultures did not yield any pathogenic organisms.

His most recent pulmonary function tests were consistent with mild obstructive lung disease and a normal diffusing capacity for carbon monoxide. Pre-bronchodilator values showed an FEV₁ (forced expiratory volume in one second) of 2.92 L (82% predicted), an FVC (forced vital capacity) of 4.34 L (89% predicted), and an FEV₁/FVC ratio of 67%. Post-bronchodilator testing demonstrated a significant response, with an FEV₁ of 3.31 L (94% predicted), reflecting an absolute increase in FEV₁ of 390 mL and a 12% improvement relative to predicted.

**Figure 1:**
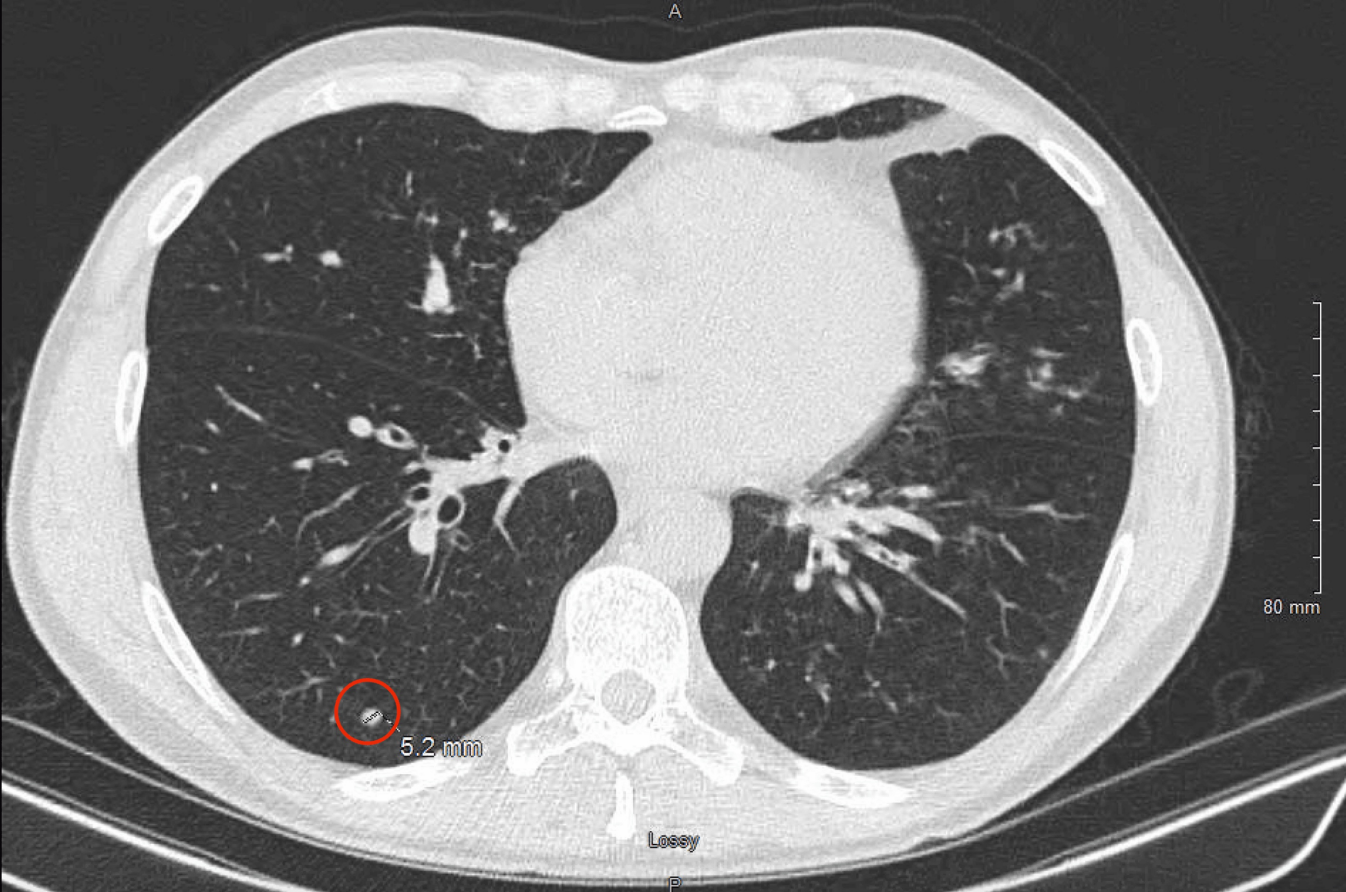
Chest CT prior to treatment initiation showing a pulmonary nodule A chest CT prior to treatment initiation shows multiple pulmonary nodules. This slice highlights a 5.2 mm nodule in the right posterior lower lobe (red circle).

**Figure 2:**
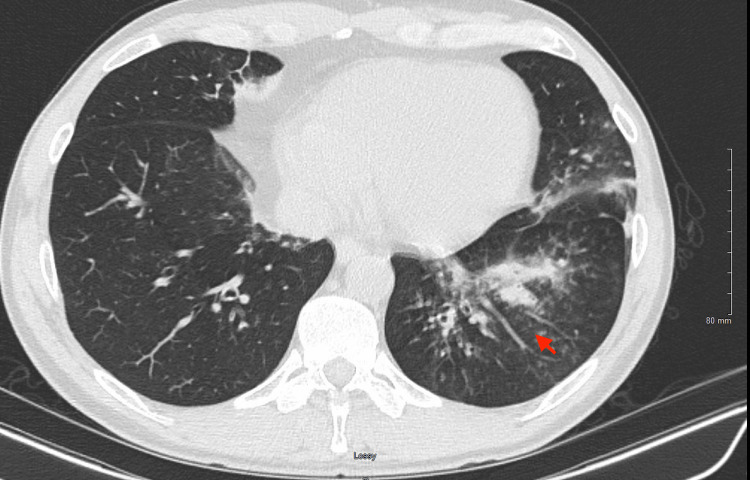
Chest CT prior to treatment initiation showing ground-glass opacity A chest CT prior to treatment initiation shows consolidation in the left lower lobe (red arrow) with associated peribronchial thickening.

Chest radiography revealed findings suggestive of airspace disease at the left lung base. Given his persistent symptoms, a chest CT was performed, which confirmed bibasilar bronchiectasis and multiple progressive pulmonary nodules, primarily in the left lower lobe, all measuring less than 1 cm. Additionally, there was mediastinal lymphadenopathy. A follow-up chest CT demonstrated progression, with increased interstitial and ground-glass opacities (GGOs) in the bilateral lower lobes. The largest nodule measured up to 5.2 mm, as shown in Figure 1, with a GGO in the left lower lung demonstrated in Figure 2.

The unexplained progression of radiographic findings and persistent symptoms prompted bronchoscopy to increase diagnostic yield. Thick, tenacious secretions were found in the airways, and the mucosa of the left lower lobe exhibited inflammation. Cultures from BAL (bronchoalveolar lavage) and bronchial wash grew pan-sensitive *Pseudomonas aeruginosa*, for which he completed a five-day course of levofloxacin. Staining for acid-fast bacteria (AFB) was negative. However, the AFB broth culture grew *Nocardia* spp. Subsequently, the specimen was sent for typing and antimicrobial susceptibility. The head CT showed no brain involvement. The susceptibility profile of *Pseudomonas aeruginosa* is presented in Table 1.

**Table 1:** Antimicrobial susceptibility profile of Pseudomonas aeruginosa isolated from bronchoalveolar lavage fluid The table shows the susceptibility profile of *Pseudomonas aeruginosa*. The interpretive categories are S = susceptible, I = intermediate, and R = resistant. MIC: minimum inhibitory concentrations

Antimicrobial agent	MIC	Interpretation
Ceftazidime	2	S
Gentamicin	≤ 1	S
Levofloxacin	≤ 0.12	S
Piperacillin/tazobactam	≤ 4	S

While awaiting susceptibility results and without evidence of *Nocardia* dissemination, he was initiated on a planned six-month course of trimethoprim-sulfamethoxazole (TMP-SMX). However, during the initial seven days of treatment, he developed fever, diffuse myalgias, headaches, and a nonpruritic, generalized rash. He did not exhibit mucosal involvement, shortness of breath, or diarrhea.

He presented to the emergency department and was found to be febrile (103°F), tachycardic (116 beats per minute), and tachypneic (26 breaths per minute), with an oxygen saturation of 98% on room air.

Laboratory evaluation revealed leukopenia (WBC 2.3 × 10⁹/L; reference range: 4.0-11.0 × 10⁹/L), thrombocytopenia (platelets 80 × 10⁹/L; reference range: 150-400 × 10⁹/L), elevated liver transaminases (AST 580 U/L, ALT 474 U/L; reference range: 10-44 U/L), hyperbilirubinemia (total bilirubin 1.4 mg/dL; reference range: 0.2-1.0 mg/dL), elevated alkaline phosphatase (227 U/L; reference range: 25-165 U/L), and hyponatremia (sodium 125 mmol/L; reference range: 135-145 mmol/L). Other tests, including lactic acid, were within normal limits. The constellation of symptoms and laboratory findings was consistent with an idiosyncratic adverse reaction to TMP-SMX, necessitating hospitalization. TMP-SMX was subsequently discontinued. Methylprednisolone and diphenhydramine were administered, leading to rapid improvement of the rash. His liver function tests gradually normalized, and a hepatitis viral panel was negative.

He was switched to an extended course of intravenous ceftriaxone while awaiting the *Nocardia* sensitivity report and ultimately received ceftriaxone for a total of four weeks. Partial 16S rRNA sequencing of the broth isolate was a 99.8% match to the *Nocardia nova* type strain, susceptible to TMP-SMX, linezolid, imipenem, amikacin, ceftriaxone, and clarithromycin. He was discharged with a six-month course of clarithromycin. The susceptibility profile of *Nocardia nova* is presented in Table 2.

**Table 2:** Susceptibility profile of Nocardia nova The table presents the antimicrobial susceptibility profile of *Nocardia nova* isolated from bronchoalveolar lavage. The interpretive categories are S = susceptible, I = intermediate, and R = resistant. TMP-SMX: trimethoprim-sulfamethoxazole; MI: minimum inhibitory concentration

Antibiotic	MIC	Interpretation
TMP-SMX	≤ 0.25/4.75	S
Linezolid	≤ 1	S
Ciprofloxacin	> 4	R
Imipenem	≤ 2	S
Moxifloxacin	4	R
Augmentin	64/32	R
Amikacin	≤ 1	S
Ceftriaxone	8	S
Doxycycline	4	I
Minocycline	2	I
Tobramycin	16	R
Clarithromycin	≤ 0.06	S

**Figure 3:**
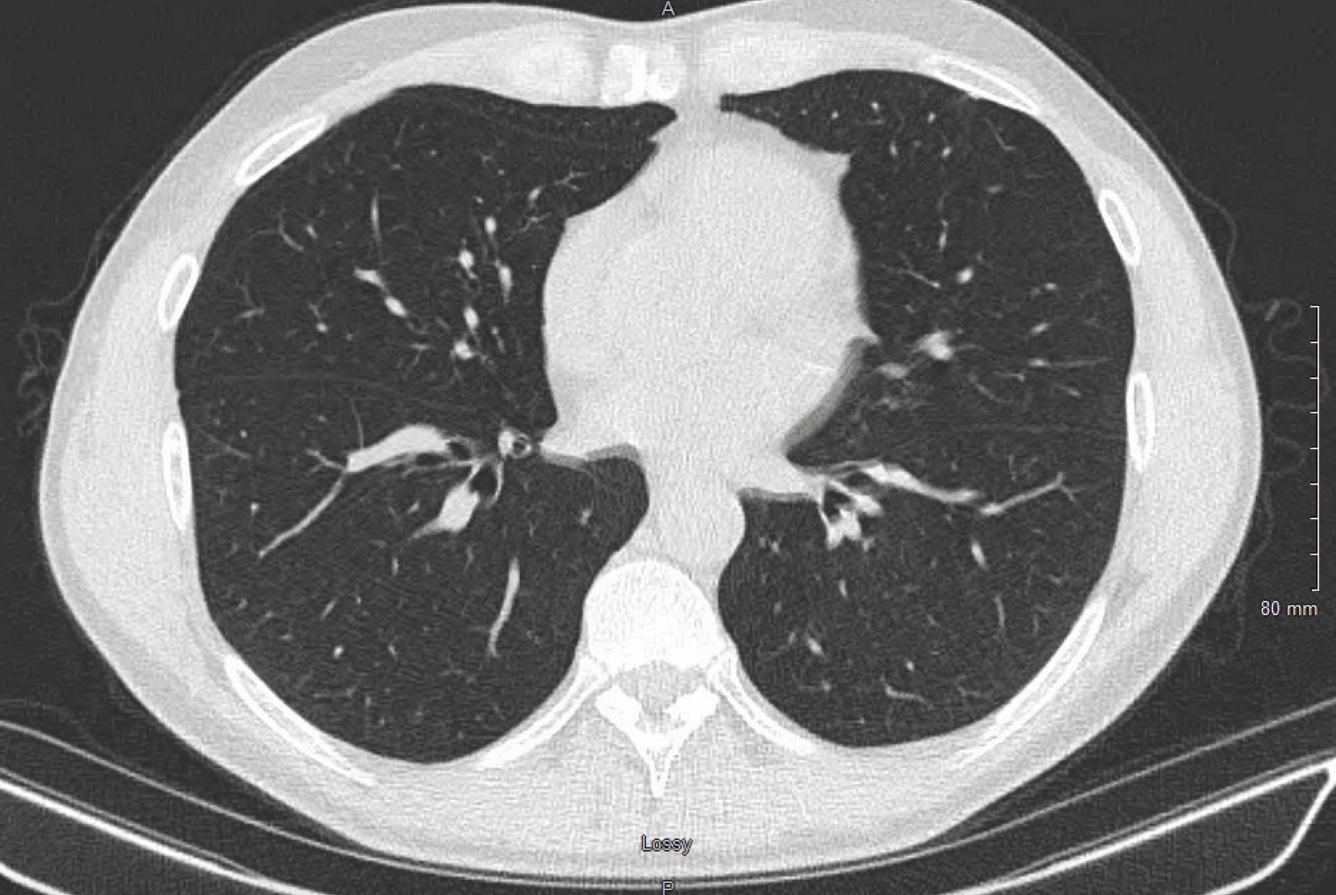
Chest CT after completion of antibiotic treatment A chest CT after the completion of antibiotic treatment demonstrates marked improvement compared to the prior study, with resolution of nodularity and ground-glass opacities in both lungs. No new nodules are identified.

A follow-up chest CT at six months showed marked improvement compared to the initial study, with reduced lymphadenopathy and resolution of most nodular areas, including the 5.2 mm nodule, and GGOs in both lungs. His cough resolved, and he has remained free of asthma symptoms. The patient continues to be followed in the pulmonary clinic. Figure 3 illustrates the post-treatment CT, demonstrating significant improvement with resolution of nodularity and GGOs in both lungs.

## Discussion

The clinical course of nocardiosis can be indolent and may mimic other infectious or non-infectious respiratory diseases, making diagnosis and treatment challenging. In this case, the patient's prolonged history of progressive productive cough, along with CT findings of mediastinal lymphadenopathy, nodules, and progressive GGOs, prompted a broad differential, including asthma exacerbations, bronchitis, community-acquired pneumonia, allergic bronchopulmonary aspergillosis, sarcoidosis, tuberculosis, non-tuberculous mycobacterial infection, and malignancy. Initial suspicion for nocardiosis was low.

Aside from well-controlled T1DM and a decreased IgG3 level, our patient had no risk factors for overt immunosuppression. While nocardiosis is typically considered an opportunistic infection in immunocompromised individuals, up to one-third of cases occur in immunocompetent hosts [1]. To date, no literature has directly linked isolated IgG3 deficiency to nocardiosis. IgG3 deficiency, though not a generalized immunosuppressive state, impairs antibody responses to polysaccharide antigens, increasing susceptibility to recurrent respiratory infections [6]. Since *Nocardia* species contain immunologically active polysaccharides, this raises the possibility that IgG3 deficiency may contribute to infection risk in affected individuals [7]. It is also worth noting that available evidence suggests that asthma is a predisposing underlying respiratory condition for pulmonary nocardiosis in immunocompetent individuals, particularly when associated with corticosteroid use or structural lung disease [9].

In respiratory nocardiosis, specimens are obtained from sputum, lavage, and biopsies [10]. For cutaneous nocardiosis, tissue biopsies are preferred for culture [11]. In all cases of pulmonary and disseminated nocardiosis, cerebral imaging is needed to rule out insidious CNS disease [12]. Our patient's nodularity with progressive GGOs on chest CT prompted diagnostic bronchoscopy. Given the clinical difficulties in diagnosing nocardiosis, up to 44% of pulmonary infections require an invasive procedure, including bronchoscopy, lung biopsy, or thoracocentesis, to establish the diagnosis of nocardiosis [3]. Therefore, this case establishes the importance of a procedural modality such as bronchoscopy for aiding diagnosis.

Our case also highlights the importance of using species typing to guide antimicrobial selection [5]. Therapeutics for nocardiosis depend on the extent of organ involvement, and empirical therapy should be commenced. However, guidance on the selection and duration of antibiotics is largely derived from retrospective studies, including observational studies, animal studies, in vitro susceptibility profiles, and expert opinion, rather than randomized controlled trials. Sulfonamides, especially in the form of TMP-SMX, are often favored, given extensive experience in their treatment application. Our case's severe drug reaction to TMP-SMX led to its discontinuation. A switch to linezolid was considered, but given leukopenia and thrombocytopenia, there was a concern about further progression to pancytopenia. He was transitioned to ceftriaxone while awaiting specialized reference laboratory type and susceptibility reporting. However, due to species-specific resistance, third-generation cephalosporins have exhibited variable activity against different *Nocardia* spp. isolates [11]. Therefore, typing and susceptibility testing are recommended, and a lack of them risks failure of initial treatment. Our case, an infection of *Nocardia nova*, was susceptible to both ceftriaxone and clarithromycin. The latter was chosen as the oral antibiotic of choice for extended treatment, and the patient was able to achieve both clinical and radiographic remission.

Patients with nocardiosis who were immunocompromised typically experienced more severe disease and significantly higher mortality compared to those who were not immunocompromised [13]. The patient, who was previously healthy and without overt immunocompromise, tobacco use, or occupational/environmental exposures, experienced a favorable outcome.

## Conclusions

We present a case of a patient with recurrent sinus infections and poorly controlled asthma-like symptoms, including chronic cough. Despite treatment optimization for asthma and administration of appropriate antibiotics, the patient's cough progressed. The chest CT demonstrated bronchiectasis, nodules, and lymphadenopathy. Finally, a bronchoscopy with BAL unveiled the culprit, *Nocardia* infection. While cellular immunodeficiency is a known risk factor for nocardiosis, isolated IgG3 deficiency has not been established as one. Asthma, especially with corticosteroid treatment or structural lung disease, is a recognized predisposing respiratory condition in nocardiosis. Given the nonspecific clinical manifestation, the diagnosis of pulmonary nocardiosis requires a high index of suspicion, especially for patients without overt immunosuppression, and may necessitate the use of procedural modalities such as bronchoscopy. Typing and susceptibility results provide the key to therapeutic success.
